# Dysregulation of Resolvin E1 Metabolism and Signaling in a Light-Damage Model of Age-Related Macular Degeneration

**DOI:** 10.3390/ijms24076749

**Published:** 2023-04-04

**Authors:** Annamaria Tisi, Giulia Carozza, Alessandro Leuti, Rita Maccarone, Mauro Maccarrone

**Affiliations:** 1 Department of Biotechnological and Applied Clinical Sciences, University of L’Aquila, 67100 L’Aquila, Italy; 2 Department of Medicine, Campus Bio-Medico University of Rome, 00128 Rome, Italy; 3 European Center for Brain Research (CERC)/Santa Lucia Foundation IRCCS, 00143 Rome, Italy

**Keywords:** bioactive lipids, SPMs, RvE1, age-related macular degeneration, light damage

## Abstract

Resolvin E1 (RvE1) is an eicosapentaenoic acid-derived lipid mediator involved in the resolution of inflammation. Here, we investigated whether RvE1 alterations may occur in an animal model of age-related macular degeneration (AMD). To this end, Sprague Dawley albino rats underwent light damage (LD), and retinas and serum were analyzed immediately or seven days after treatment. Western blot of retinas showed that the RvE1 receptor ChemR23 and the RvE1 metabolic enzymes 5-LOX and COX-2 were unchanged immediately after LD, but they were significantly up-regulated seven days later. Instead, the RvE1 receptor BLT1 was not modulated by LD, and neither was the RvE1 degradative enzyme 15-PGDH. Moreover, ChemR23, 5-LOX, COX-2 and BLT1 were found to be more expressed in the inner retina under all experimental conditions, as observed through ImageJ plot profile analysis. Of note, amacrine cells highly expressed BLT1, while ChemR23 was highly expressed in the activated microglia of the outer retina. ELISA assays also showed that LD rats displayed significantly higher circulating levels and reduced retinal levels of RvE1 compared to controls. Altogether, our data indicate that RvE1 metabolism and signaling are modulated in the LD model, suggesting a potentially relevant role of this pathway in AMD.

## 1. Introduction

The termination of inflammatory events has been considered a passive process for a long time, possibly triggered by a gradual dissipation of pro-inflammatory events. With the discovery of pro-resolving mediators biosynthesized from ω-3 (n-3) essential fatty acids, to date it is increasingly evident that the resolution of inflammation is instead an active process, which is turned on to limit acute inflammation and to avoid the progression towards chronic inflammation [1]. In this context, “resolvins”, endogenous lipids belonging to a class of bioactive lipids collectively known as “specialized pro-resolving mediators” (SPMs), have emerged as key regulators of the resolution of inflammation [2]. Resolvins are synthesized from ω-3 (n-3) PUFAs through a series of metabolic reactions, which involve mainly cyclooxygenase-2 (COX-2) and various lipoxygenase (LOX) isozymes, and are then degraded by enzymes such as 15-hydroxyprostaglandin dehydrogenase (15-PGDH) [1,2]. SPMs exert their biological activity through binding to selective G protein coupled receptors (GPCRs) [2]. Within SPMs, E-series resolvins (RvE1, RvE2, RvE3) are derived from eicosapentaenoic acid (EPA), while D-series resolvins (RvD1, RvD2, RvD3, RvD5 and RvD6) are derived from docosahexaenoic acid (DHA) [1]. Incidentally, it can be recalled that the term “resolvins” was introduced by Serhan and colleagues in 2002 to describe the potential of these mediators to downregulate leukocyte exudation and regulate the resolution of inflammation [3]. Accordingly, several studies have demonstrated that alterations of metabolism and signaling of resolvins are involved in multiple diseases characterized by chronic inflammation, and that resolvins administration represents a promising therapeutic approach for such diseases [2]. Noteworthy, very little is known about the role of resolvins in retinal neurodegenerative diseases [4]. For instance, resolvin D1 (RvD1) is the only resolvin investigated so far in in vivo models of retinal diseases, where its administration protects against retinal degeneration when using the endotoxin-induced mouse model of uveitis [5,6]. A few additional in vitro studies also indicated that both RvD1 and RvE1 may exert potent anti-inflammatory effects in retinal cells [7,8,9]. Remarkably, despite the lack of information about its mechanism of action in the retina, an analogue of RvE1 (RX-10045) is currently being used in clinical trials (clinical trial identifiers NCT02329743, NCT00799552) to treat ocular inflammatory diseases at the eye’s surface [10]. This makes RvE1 of particular interest from a translational point of view for ophthalmic applications, and encourages the study of RvE1 metabolism/signaling also in diseases of the neuroretina that are characterized by inflammation. Thus, understanding molecular details regarding the possible involvement of RvE1 in retinal degeneration seems urgent. In this context, over the last decade, evidence has been accumulated about causal correlations between chronic inflammation and age-related macular degeneration (AMD) [11]—the leading cause of blindness in developed countries in aged people (>55 years) [12]—suggesting that resolvins’ alterations may occur in AMD much alike other neurodegenerative diseases [2,3]. In AMD, chronic inflammation is mainly triggered by ageing, genetic predisposition, environmental factors or epigenetic changes, leading to microglia activation and gliosis, and the up-regulation of pro-inflammatory cytokines and chemokines (i.e., IL-6, IL-8, TNF, IL-1α, IL-1β, MCP-1, MCP-2, CX3CL1, IGF–IGFR and colony-stimulating factors) [11]. Of note, genetic polymorphisms that predispose to AMD often involve inflammatory-associated genes, such as the complement factor H (CFH) and other genes of the complement cascade [13].

Altogether, the existing literature indicates a major role of inflammation in the development of AMD and supports the hypothesis that the resolution of inflammation may be altered. However, no data are available yet on the possible involvement of resolvins in AMD. With the aim of filling this knowledge gap, here we investigated the possible alterations of RvE1 metabolism and signaling in an animal model of AMD. 

## 2. Results

### 2.1. Retinal Light Damage up-Regulates RvE1 Metabolic Enzymes

The main biosynthetic enzymes of RvE1 are COX-2 and 5-LOX, which generate it from EPA [1,2]. Firstly, COX-2 converts EPA into 18(R)-HpEPE, which is then converted into RvE1 by 5-LOX. RvE1 degradation occurs via 15-hydroxyprostaglandin dehydrogenase (15-PGDH), which terminates its biological activity [14]. Therefore, to obtain insights into RvE1 metabolism, protein levels of COX-2, 5-LOX and 15-PGDH were quantified in the retinas of the light damage (LD) model of AMD. 

#### 2.1.1. COX-2 

**Figure 1:**
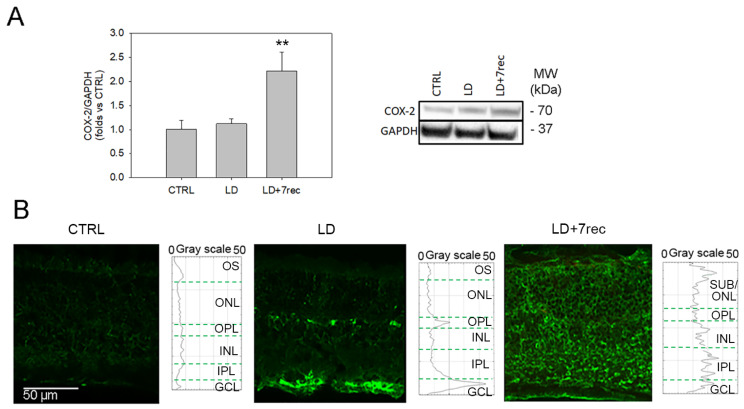
COX-2 analysis. (**A**) Western blot analysis of COX-2 in eye cup samples of all the experimental conditions. Statistical analysis was one-way ANOVA test followed by Tukey test. Data are shown as mean ± SE (*n* = 6). ** *p* < 0.01 versus CTRL. The whole Western blot is available as Supplementary Figure S1. (**B**) Confocal images of retinal cryosections immunolabelled for COX-2 (green) acquired at the central dorsal area of the retina. The graphs show the plot profile fluorescence intensity of COX-2 through the retinal layers. CTRL (Control); LD (Light damage); LD + 7rec (Light damage + 7 days of recovery); OS (outer segments); ONL (Outer nuclear layer); OPL (outer plexiform layer); INL (Inner nuclear layer); IPL (inner plexiform layer); GCL (Ganglion cell layer); ONL/SUB (outer nuclear layer/subretina).

COX-2 protein levels were not modulated immediately after LD (LD group); yet, they were significantly increased seven days after injury (LD + 7rec group) compared to healthy animals (CTRL group) (*p* = 0.009) (Figure 1A). Immunofluorescence staining of retinal cryosections from the same experimental groups was then used to localize COX-2 throughout the retinal layers; moreover, COX-2 expression was quantified as mean of fluorescence intensity (MFI) through plot profile analysis. As shown in Figure 1B, the CTRL group expresses COX-2 in all retinal layers. After LD, the COX-2 signal was increased in the outer plexiform layer (OPL) and ganglion cell layer (GCL), while it was decreased in the photoreceptors’ outer segments (OS). The different expression of COX-2 through the retinal layers of CTRL and LD groups may also explain why no differences could be observed by means of Western blot, which indeed pools together COX-2 proteins of all parts of the retina. COX-2 immunostaining on LD + 7rec retinas also increased in all retinal layers compared to CTRL, consistently with the Western blot result.

#### 2.1.2. 5-LOX

**Figure 2:**
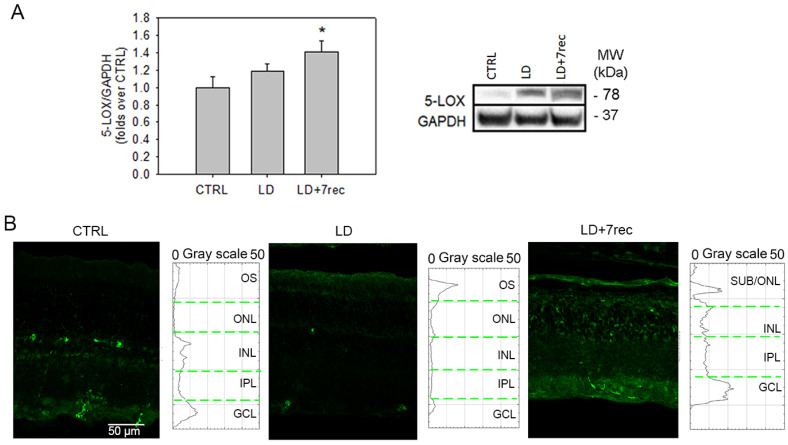
5-LOX analysis. (**A**) Western blot analysis of 5-LOX in eye cup samples of all the experimental conditions. Statistical analysis was a one-way ANOVA test followed by Tukey test. Data are shown as mean ± SE (*n* = 12). * *p* < 0.05 versus CTRL. The whole Western blot is available as Supplementary Figure S2. (**B**) Confocal images of retinal cryosections immunolabelled for 5-LOX (green) acquired at the central dorsal area of the retina. Scale bar: 50 µm. The graphs show the plot profile fluorescence intensity of 5-LOX throughout the retinal layers. CTRL (Control); LD (Light damage); LD + 7rec (Light damage + 7 days of recovery); OS (outer segments); ONL (Outer nuclear layer); OPL (outer plexiform layer); INL (Inner nuclear layer); IPL (inner plexiform layer); GCL (Ganglion cell layer).

Similarly to COX-2, 5-LOX analysis demonstrated an increase in the LD + 7rec group compared to the CTRL (Figure 2). Specifically, the Western blot technique (Figure 2A) showed an increase in 5-LOX protein levels 7 days after the injury, compared to the CTRL (*p* = 0.042), while no differences were found between the LD and CTRL groups. The immunofluorescence staining and plot profile analysis (Figure 2B) showed that 5-LOX expression was increased in the OS of the LD group, alongside a reduction in the inner retina compared to the CTRL. This “shift” of 5-LOX expression may explain the similarity in protein levels detected by the Western blot technique. Instead, 5-LOX fluorescence increased through all retinal layers in the LD + 7rec group compared to the CTRL, consistently with the Western blot analysis. 

#### 2.1.3. 15-PGDH

A previous study suggested that 15-PGDH activity is not measurable in ocular tissues [15]. Consistently, here, 15-PGDH expression could not be detected in rat retinas under experimental conditions. To rule out any failure in technical procedures, a Western blot was performed on a rat liver as a positive control, where 15-PGDH was highly detectable (Supplementary Figure S3). 

### 2.2. ChemR23, the Selective RvE1 Receptor, Is Modulated by Retinal Light Damage

**Figure 3:**
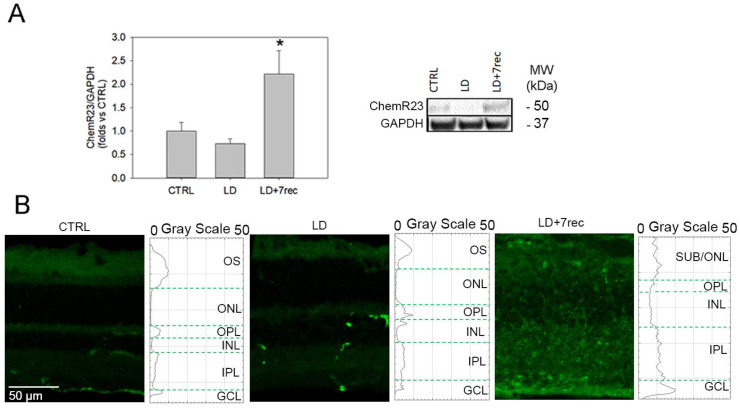
ChemR23 analysis. (**A**) Western blot analysis of ChemR23 in eye cup samples of all the experimental conditions. Statistical analysis was one-way ANOVA test followed by Tukey test. Data are shown as mean ± SE (*n* = 6). * *p* < 0.05 versus CTRL. The whole Western blot is available as Supplementary Figure S4. (**B**) Confocal images of retinal cryosections immunolabelled for ChemR23 (green) acquired at the central dorsal area of the retina. Scale bar: 50 µm. The graphs show the plot profile fluorescence intensity of ChemR23 throughout the retinal layers. CTRL (Control); LD (Light damage); LD + 7rec (Light damage + 7 days of recovery); OS (outer segments); ONL (Outer nuclear layer); OPL (outer plexiform layer); INL (Inner nuclear layer); IPL (inner plexiform layer); GCL (Ganglion cell layer).

Bioactive lipids exert their biological activity through the binding to specific receptors. The chemerin receptor 23 (ChemR23), a G-protein coupled receptor (GPCR), selectively binds RvE1, thus activating the pro-resolving phenotype typically triggered by RvE1 [16]. Here, we quantified ChemR23 protein levels by Western blot (Figure 3A) in eye cup samples from all experimental groups. A similar expression of ChemR23 was observed in the CTRL and LD groups, while it was significantly up-regulated in the LD + 7 rec group compared to the CTRL (*p* = 0.028).

The immunofluorescence staining and plot profile analysis of retinal cryosections (Figure 3B) was consistent with the Western blot data. Specifically, the CTRL and LD groups showed similar ChemR23 levels, while ChemR23 fluorescence increased through all retinal layers compared to the CTRL.

### 2.3. Expression of ChemR23 by Resting and Activated Retinal Microglia

Activated microglia play a pivotal role in the degenerative events of AMD [17]. Likewise, in the LD model of AMD, resting microglia activate and migrate to the outer retina (ONL/subretina), contributing to the chronic inflammatory events occurring at the site of the injury [18,19,20,21]. Recently, in a neurodegenerative/neuroinflammatory disorder such as Alzheimer’s disease, it has been demonstrated that activated microglia are recruited to the site of injury via ChemR23 activation [22,23].

**Figure 4:**
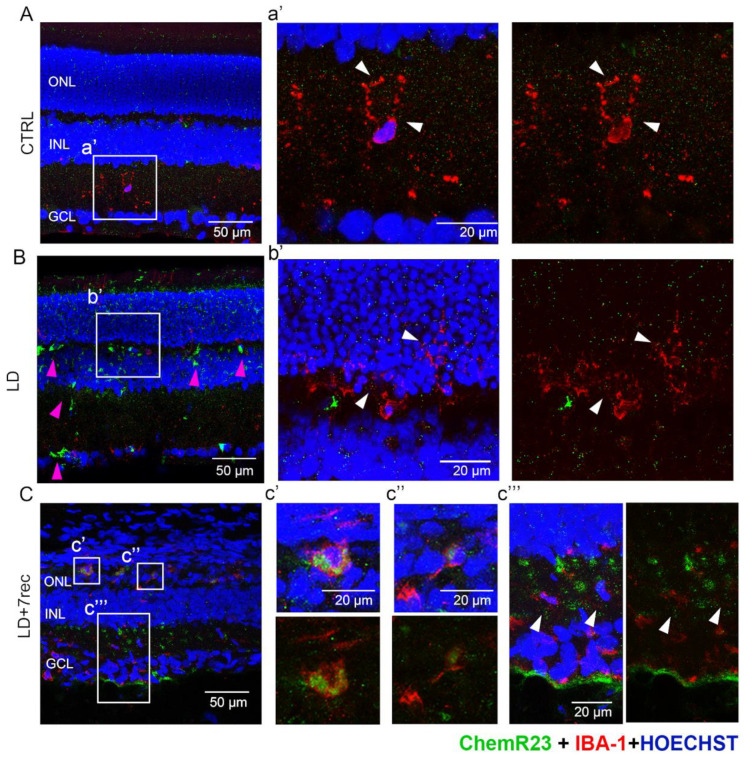
Double immunostaining for IBA-1 and ChemR23. (**A**–**C**) Confocal images of retinal cryosections labelled for IBA-1 (red) and ChemR23 (green), and counterstained with bisbenzimide nuclear dye (blue) of (**A**) CTRL, (**B**) LD and (**C**) LD + 7rec retinas. Scale bar: 50 µm. High magnifications (**a’**,**b’**,**c’**,**c’’**,**c’’’**), scale bar 20 µm. The white arrows show detailed localization of ChemR23 in IBA-1 (+) cells. The pink arrows indicate increased ChemR23 signal in the LD group not co-localizing with IBA-1 (+) cells. CTRL (Control); LD (Light damage); LD + 7rec (Light damage + 7 days of recovery); ONL (Outer nuclear layer); INL (Inner nuclear layer); GCL (Ganglion cell layer).

We therefore hypothesized that also in our AMD model, ChemR23 may be involved in microglia activation. On this basis, we performed double immunostaining for ChemR23 and the selective microglia marker IBA-1 in retinal cryosections of all experimental groups (Figure 4). 

In the healthy retina, microglia are in a resting state and are only present in the inner retina [18], where ChemR23 was weakly expressed mostly as puncta (Figure 4A,a’). After LD, microglia starts to activate, proliferate and infiltrate in the outer retina [18]. In addition, at this time point, ChemR23 puncta were detectable in microglia invading the ONL (Figure 4B,b’). Interestingly, increased ChemR23 expression was detectable in the LD group (Figure 4, pink arrows), but not all the signals co-localized with microglia, suggesting that ChemR23 is up-regulated in other retinal cells. Seven days after LD, multiple activated microglia migrated to the ONL and subretinal space [18], and also displayed a different morphology whereby they switched from a ramified to a more ameboid (with reduced branches size) phenotype, as shown in Figure 4C. In those cells, ChemR23 was widely expressed in the cell bodies (Figure 4c’,c’’), whereas ChemR23 puncta were present in the resting microglia of the inner retina of the LD + 7rec group (Figure 4c’’’). Supplementary Figure S6 reports surface plot profiles of resting and activated microglia, showing different morphological features and ChemR23 expression. 

### 2.4. The Non-Selective RvE1 Receptor BLT1 Is not Modulated by Retinal Light Damage

**Figure 5:**
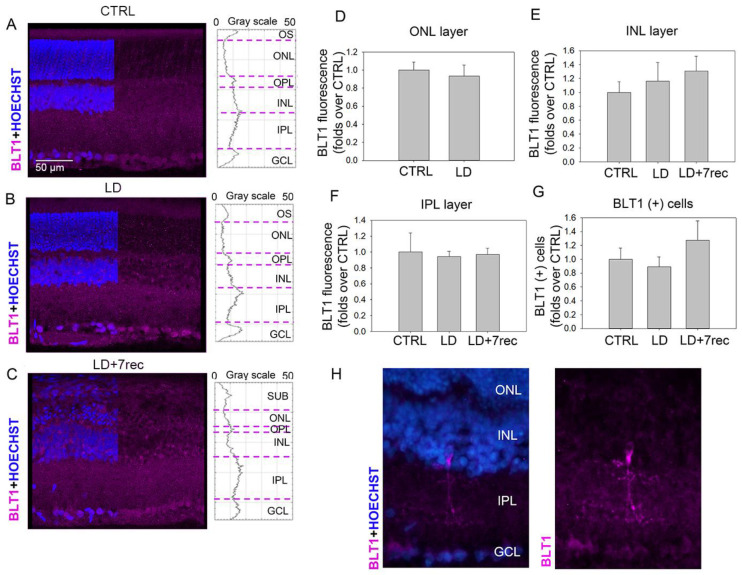
BLT1 analysis. (**A**–**C**) Confocal images and plot profile of fluorescence intensity of retinal cryosections from CTRL, LD and LD + 7rec groups, respectively, immunolabelled for BLT1 (pink). Nuclei were stained with Bisbenzimide (blue). The images refer to the central dorsal area of the retina. Scale bar: 50 µm. BLT1 fluorescence intensity in (**D**) the ONL; (**E**) the INL; (**F**) in the IPL. (**G**) Quantitative analysis of BLT1 (+) cells from the superior to the inferior edge of retinal cryosections. Data are shown as mean ± SE (*n* = 4). (**H**) High magnification of random BLT1 (+) cells (pink). CTRL (Control); LD (Light damage); LD + 7rec (Light damage + 7 days of recovery); OS (outer segments); ONL (Outer nuclear layer); OPL (outer plexiform layer); INL (Inner nuclear layer); IPL (inner plexiform layer); GCL (Ganglion cell layer).

Recent studies indicate that RvE1 acts as an antagonist of the leukotriene B_4_ receptor 1 (BLT1) [16]. Therefore, the possible modulation of BLT1 by retinal light damage was also investigated. Due to the lack of anti-BLT1 antibodies suitable for Western blot analysis in rats, analyses were focused on the fluorescence intensity of BLT1 in retinal cryosections (Figure 5). 

In CTRL animals, BLT1 was widely expressed in the retina. The localization was not different in LD animals even 7 days after the damage. Moreover, the BLT1 plot profiles were similar under all conditions (Figure 5A). Consistently, BLT1 fluorescence intensity was similar in the ONL (Figure 5B), INL (Figure 5C) and IPL (Figure 5D) layers of all experimental groups. It should be stressed that BLT1 fluorescence could not be quantified in the ONL of LD + 7rec retinas because degenerated tissue in that layer was characterized by autofluorescence, which may bias the analysis due to cellular debris [24].

Intriguingly, sporadic cells in the inner retina were densely positive to BLT1 (Figure 5H). The morphology and localization of those cells are typical of amacrine cells, suggesting that BLT1 was highly expressed in such a specific cell type of the retina. It was thus investigated whether a difference in the number of BLT1 (+) cells existed between CTRL and LD animals, and no significant differences were found between the experimental groups (Figure 5G). Altogether, the BLT1 analysis indicates that this receptor was not modulated by light damage. 

### 2.5. RvE1 Levels Are Affected by Light Damage

In order to better understand the modifications of RvE1 metabolism/signaling, the levels of RvE1 were quantified in both retinal samples and serum from all experimental groups through an ELISA assay.

The retinal levels of RvE1were extremely low; hence, it was not feasible to quantify them in individual samples. Therefore, five retinas from the same experimental group were pooled together, and it could be shown that RvE1 retinal levels were ~520 pg/mL in the CTRL; LD dramatically reduced the RvE1 levels to ~100 pg/mL and ~170 pg/mL, in the LD and LD + 7rec groups, respectively. 

**Figure 6:**
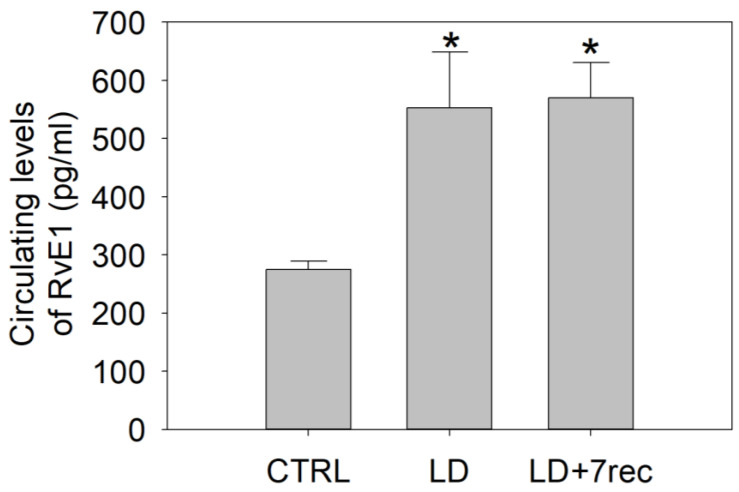
Quantification of RvE1 in the serum of LD rats. ELISA assay was performed on serum samples from all experimental groups. Statistical analysis was performed by one-way ANOVA test followed by Tukey test. Data are shown as mean ± SE (*n* = 4). * *p* < 0.05 versus CTRL. CTRL (Control); LD (Light damage); LD + 7rec (Light damage + 7 days of recovery).

Circulating RvE1 levels showed an opposite trend (Figure 6), with RvE1 being significantly increased in the serum of LD (*p* = 0.031) and LD + 7rec (*p* = 0.023) rats compared to the CTRL. 

## 3. Discussion

In the present study, we investigated for the first time RvE1 metabolism and signaling in a widely used and well characterized model of AMD, known as retinal light damage (LD), which is based on the induction of retinal degeneration through the exposure to high intensity light [19,25,26,27]. We hypothesized that RvE1 dysregulation may occur in AMD as one of the mechanisms at the basis of chronic inflammation typically associated with the disease. Overall, the present findings showing that RvE1 metabolism and signaling are altered in the LD model support that the resolution of inflammation may play a role in AMD pathogenesis. 

### 3.1. Metabolism of RvE1 Is Altered in the LD Model

**Figure 7:**
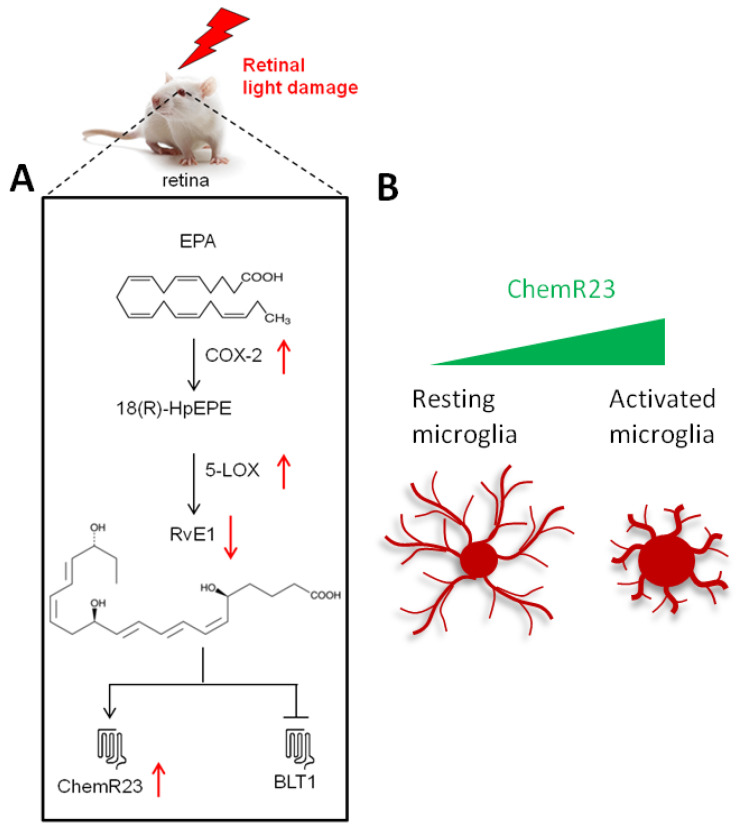
Schematic illustration of the main findings of the study. The scheme summarizes the results obtained from the retinas 7 days after LD (LD + 7rec group). (**A**) Effects of light damage (LD) on RvE1 metabolism and signaling in the rat retinas; the black lines indicate RvE1 metabolism and signaling, while the red arrows indicate the events induced by LD. (**B**) Schematic cartoon illustrating the different expressions observed in retinal resting and activated microglia.

RvE1 metabolism and signaling were investigated in the retinas of LD rats at two critical time points: (i) immediately after LD, that is when the peak of apoptosis occurs in the photoreceptors and retinal pigment epithelial (RPE) cells of the outer retina, the blood–retinal barrier (BRB) starts to disaggregate, and the degenerative mechanisms are induced [28,29]; and (ii) 7 days after LD, that is when photoreceptor and RPE atrophy occur in a limited area of the dorsal retina (known as hotspot) alongside BRB breakdown, functional impairment, gliosis, accumulation of autofluorescent cell debris, neovascularization, and additional molecular/degenerative events typical of an advanced AMD form [24,26,27,29]. In our study, the main RvE1 metabolic alterations were found 7 days after LD—as summarized in Figure 7—when the selective RvE1 receptor ChemR23 was also significantly up-regulated compared to healthy retinas. It is noteworthy that although no differences in the protein levels of RvE1 metabolic enzymes could be detected immediately after LD, a different localization occurred in the retinas according to the plot profiles graphs (Figure 1B and Figure 2B), suggesting that alterations in RvE1 metabolism/signaling are also taking place immediately after LD. In line with this, RvE1 levels were decreased in the retinas both immediately and 7 days after the photopic injury, while circulating levels of RvE1 in both groups increased compared to CTRL. Several considerations should be taken into account to explain these observations:

(i) COX-2 and 5-LOX can mediate also the metabolism of bioactive lipids beyond RvE1, such as arachidonic acid-derived eicosanoids and additional SPMs [1,2];
(ii) the known inactivating enzyme of RvE1, 15-PGDH [30], is not expressed by retinal cells, suggesting that independent (and largely unknown) mechanisms may take place to remove and inactivate RvE1, possibly by transportation to the choriocapillaris via the BRB. Yet, retinal lipid metabolism still needs to be fully elucidated [31];
(iii) LD leads to BRB breakdown [29], and therefore the passage of substances inside/outside the retina likely perturbs its fine regulation;
(iv) circulating RvE1 levels may be increased as an adaptive mechanism, able to respond to the stress due to the LD procedure and blindness development, or may be the result of the stimulation of additional non-retinal photoreceptors [32,33], without necessarily reflecting the ocular condition [34].

### 3.2. ChemR23 May Be Involved in the Recruitment of Retinal Activated Microglia

ChemR23 is the best known selective receptor for RvE1. The RvE1/ChemR23 axis triggers multiple pro-resolving events, such as the regulation of vascular inflammation, boosting of macrophage phagocytosis and dampening of neuroinflammation [14]. Here, we show for the first time the localization of ChemR23 in the retina and provide unprecedented evidence of its involvement in the pathological events occurring in AMD. Firstly, we demonstrate that ChemR23 is expressed through all retinal layers, and its protein levels are up-regulated by LD with a major impact on activated microglia (Figure 7). Indeed, microglia are the sentinels of the central nervous system and are recruited to the site of injury with the goal of contrasting noxious events. However, it is widely accepted that in almost all neurodegenerative diseases, microglia fail to exert a neuroprotective role, and rather become active players in chronic inflammation and disease progression [35]. Of note, recent studies in Alzheimer’s disease have shown that activated microglia are recruited to the site of injury via ChemR23 activation [22,23], suggesting a major role of this receptor in the activation and migration of microglia in neurodegenerative/neuroinflammatory disorders. Based on our results, it can be speculated that the same may occur in the retinal microglia of the LD model, thus representing an additional pathogenic mechanism in AMD. Indeed, we found that activated microglia support a strong ChemR23 signal in the hotspot region. Further studies are needed to confirm this hypothesis by means of functional approaches. In this context, it is important to note that ChemR23 has another endogenous ligand, known as Chemerin. The latter is a protein that regulates inflammation, metabolism, and cancer [36,37]. Therefore, the recruitment of retinal microglia to the degenerating area may be mediated by Chemerin rather than RvE1. This hypothesis is consistent with the reduced RvE1 retinal levels upon LD, which reflects the inflammatory events typical of this type of retinal damage [18,20,21,38,39].

### 3.3. The Expression of RvE1 Receptors in the Neuroretina Suggests a Possible Implication in Retinal Function

Several studies indicate that bioactive lipids are involved in functional events and synaptic plasticity of the central nervous system [40,41]. For instance, in the retina, recent studies have shown that endocannabinoids, a major class of bioactive lipids, control synaptic transmission [42,43]. Based on the localization of RvE1 receptors ChemR23 and BLT1 that are shown here to be expressed in the inner retina, it can be hypothesized that these receptors sustain retinal synaptic transmission. For instance, ChemR23 was found to be strongly expressed in certain retinal cells (different from microglia) in the LD and LD + 7rec groups, suggesting an implication of retinal neurons or endothelial cells. In this context, the BLT1 localization in the amacrine cells is of particular interest, as the amacrine cells are involved in the signal transmission from photoreceptors to ganglion cells. Amacrine cells form a heterogeneous and complex retinal population, whose function has been understood only in a few primate and non-primate retinas [44]. Hence, the localization of BLT1 in amacrine cells shown here appears of particular interest, and paves the way for further investigations into its molecular details and functional implications. Likewise, a detailed investigation using specific amacrine cell markers would be useful to further investigate which amacrine cell sub-populations are involved. 

## 4. Materials and Methods

### 4.1. Animals 

All animal experiments were conducted according to the ARVO statement for the use of animals in ophthalmic and vision research and were authorized by the Italian Ministry of Health (authorization number 20/2022-PR). 

The light damage (LD) model of AMD was used to investigate RvE1 alterations in the retina. LD resembles multiple features of the AMD phenotype from early to advanced stages of the disease, including RPE dysfunction and BRB breakdown [26,45], alongside the accumulation of lipofuscin/drusen-like debris [24], neovascularization [26,46], retinal functional impairment [18,26,47] and development of RPE/photoreceptors atrophy [26,39,47] as it occurs in late AMD. Briefly, male and female Sprague Dawley (SD) albino rats were born and raised in dim cyclic light conditions (12 h light, 12 h dark) with an environmental illumination of around 5 lux and with free access to food and water. Rats from 2 to 4 months of age were included in the experiments for LD exposure. Albino rats are highly susceptible to light, and they represent an excellent and fast model to mimic AMD pathogenesis in a short time span. In fact, 24 h of exposure to high light trigger all the events that induce the abovementioned AMD signatures and that can be highlighted seven days after the photooxidative stimulus. 

### 4.2. Retina Light Damage and Experimental Design

Animals were housed in separate Plexiglas cages with food on the floor and water in transparent plastic bottles. After dark adaptation overnight, they were exposed to an acute LD (1000 lux for 24 h) starting from 9 a.m. in order to not interfere with the circadian rhythm as already described in our previous papers [19,25,26,27]. A group of animals was euthanized immediately after LD (LD group), while another was returned to dim cyclic light settings for 7 days (LD + 7rec group) to allow retinal degeneration. Both groups were compared to healthy animals (CTRL group). 

In summary, the following experimental groups were included in the present study:

(1) CTRL: healthy animals used as control;
(2) LD: animals exposed to LD and euthanized immediately after;
(3) LD + 7rec: animals exposed to LD and euthanized 7 days thereafter.

### 4.3. Protein Extraction

Protein levels of the main RvE1 metabolic (COX-2 and 5-LOX) and degradative (15-PGDH) enzymes, and of the selective RvE1 receptor (ChemR23) were quantified through the Western blot technique on eye cup samples. Briefly, eyes were enucleated, and the anterior part (cornea and lens) was removed. The remaining tissue (eye cup) was used for protein extractions. Total proteins were extracted using a Dounce Homogenizer and a lysis buffer (50 mM Tris-HCl pH 7.5, 1% Triton X-100, 0.1% SDS, EDTA 5 Mm, Halt Protease and Phosphatase Inhibitor Cocktail, Thermo Fisher Scientific, Waltham, MA, USA and QS dH_2_O). After centrifugation (15.000 rpm for 15 min), the supernatant was collected and stored at −80 °C. A Bradford Assay (Bio-Rad Laboratories, Milan, Italy) was performed in order to quantify the protein content. The rat liver was used as a positive control for quantification of 15-PGDH. The extraction for the rat liver followed the same procedure.

### 4.4. Western Blot

Firstly, 70 µg of the protein extracts were run on a Bolt 4–12% Bis-Tris Plus (Thermo Fisher Scientific) at 200 V for 20 min. The iBlot 2 Dry Blotting System (Invitrogen IB21001) was used to transfer the proteins to PVDF membrane (Millipore, Milan, Italy). Aspecific bindings were blocked with 5% nonfat dry milk in TBST at RT for 1 h; the membranes were washed briefly and incubated with primary antibodies (for detection of COX-2, 5-LOX, 15-PGDH, ChemR23) diluted in 5% nonfat dry milk in TBST. All the primary antibodies used in this paper are summarized in Table S1 in the Supplementary Materials. Afterward, the membranes were incubated for 1 h at RT with the specific HRP-conjugated secondary antibody (anti-rabbit or anti-mouse) diluted 1:2000 in 5% nonfat dry milk in TBST. The membranes were then incubated in SuperSignal West Pico Plus (Thermo Fisher Scientific Inc.) chemiluminescent substrate and the bands were detected using a ChemiDoc XRSplus imaging system (Bio-Rad Laboratories). The optical densities of blot bands were analyzed and quantified by ImageJ (U.S. National Institutes of Health, Bethesda, MD, USA) software analysis and normalized versus Glyceraldehyde-3-Phosphate Dehydrogenase (GAPDH) as the housekeeping protein.

### 4.5. Retinal Cryosections

The eyes enucleated for morphological analyses were fixed in 4% paraformaldehyde for 6 h and then washed in 0.1 M phosphate buffered saline (PBS, pH 7.4). The cornea and lens were removed, and the remaining eye cups were cryoprotected by immersion in 30% sucrose overnight, embedded in the Tissue Tek OCT (optimum cutting temperature, Qiagen, Valencia, CA, USA) compound and frozen in liquid nitrogen. Cryosections of 20 μm thickness were made through a Leica CM1850 cryostat (GmbH, Nussloch, Germany) and collected on gelatin and poly-l-lysine-coated slides. For immunofluorescence analysis, the sections crossing the optic nerve were selected. 

### 4.6. Immunofluorescence Staining

Immunofluorescence on retinal cryosections was used to identify the localization of RvE1 metabolic/degradative enzymes and RvE1 receptors (ChemR23 and BLT1) and quantify them throughout the retinal layers. Specifically, non-specific binding sites were blocked using 5% bovine serum albumin (BSA) for 1 h at room temperature (R.T.). Sections were then incubated overnight at 4 °C with primary antibodies (for detection of COX-2, 5-LOX, 15-PGDH, ChemR23 and BLT1) (see Table S1 for further details). Sections were then incubated with secondary antibodies (anti-mouse or anti-rabbit IgG conjugated to red or green fluorescent dies) (Alexa Fluor 594 or 488; Molecular Probes, Invitrogen, Carlsbad, CA, USA) diluted 1:300 and incubated at 37 °C for 2 h. Bisbenzimide nuclear dye (Hoechst) was used to label the nuclei. Negative controls obtained by staining retinal cryosections without the primary antibodies are reported in Supplementary Figure S5. 

### 4.7. Confocal Microscopy and Images Analysis

Images of immunolabeled cryosections were acquired by using a Leica TCS SP5 (Wetlzar, Germany) or a Nikon 80i confocal microscope. The central dorsal retina was selected for the analysis, since retinal degeneration develops in that specific region called a hotspot [39]. The same parameters were set up for all the acquisitions. For the final images, ~22 planes at a distance of 0.5 µm were acquired. The fluorescence intensity of all markers was quantified through ImageJ software. Differences in immunofluorescence signals throughout the retinal layers (OS, ONL, OPL, INL, IPL, GCL) were investigated through ImageJ software and by using profile plots (range 0–50) with the corresponding grayscale intensities. For the LD + 7rec group, the outer retina was indicated as SUB/ONL (subretina/outer nuclear layer) since the degenerated tissue loses the physiological retinal architecture with the occurrence of rosettes and neovascularization from the choroid [26].

### 4.8. ELISA Assay

RvE1 levels were quantified in the retina and serum of rats from all experimental groups through an enzyme-linked immunosorbent assay (ELISA) kit (#MBS733910, MyBiosource Southern California, San Diego, CA, USA) according to manufacturer instructions. For retinal samples, the retinas were removed from the eyes immediately after sacrifice and collected at −80 °C until use. Before quantification, the retinas were homogenized in PBS according to manufacturer instructions and immediately used. A pool of 5 retinas of the same experimental group was necessary to obtain detectable levels of RvE1. For serum samples, the blood was collected from the heart, let to coagulate for 15 min at R.T. and then centrifuged at 3000 rpm at 4 °C. The supernatant was then collected and stored at −80 °C until use. Optical Density (O.D.) was determined at 450 nm using a TECAN Magellan Pro v7.4 microplate reader (Männedorf, Switzerland) and RvE1 content was quantified based on RvE1 standards (range from 0 to 2500 pg/mL) used as references.

### 4.9. Statistical Analysis

Statistical analysis was performed by one-way ANOVA followed by Bonferroni post hoc comparison. The first type error was set at 5%. The statistical analysis was conducted using the SigmaPlot 12.0 Systat software (Palo Alto, CA, USA).

## 5. Conclusions

Overall, the findings of the present study demonstrate that the metabolism and signaling of RvE1 are altered in the LD model of AMD. Moreover, we showed for the first time the localization of RvE1 receptors in the retina. This is the first evidence of the possible implication of pro-resolving lipid mediators in the pathogenesis of AMD, and represents a fundamental step forward in the understanding of the mechanisms underlying such a complex disease. Resolvin E1 may therefore represent a target for the development of new AMD therapies. 

## Data Availability

Data are contained within the article or in the Supplementary Material.

## Supplementary Materials

The following supporting information can be downloaded at https://www.mdpi.com/article/10.3390/ijms24076749/s1.
