# Supplementary material for: Dysregulation of Resolvin E1 Metabolism and Signaling in a Light-Damage Model of Age-Related Macular Degeneration
Source: Int J Mol Sci. 2023 Apr 4;24(7):6749. doi: 10.3390/ijms24076749 (PMC10095591; doi:10.3390/ijms24076749)
Supplement: Supplementary file 1 [file ijms-24-06749-s001.zip › ijms-2269410-supplementary.pdf]

Supplementary material

Dysregulation of resolvin E1 metabolism and signaling in a light-damage model of age-related macular degeneration

Annamaria Tisi [1], Giulia Carozza [1], Alessandro Leuti[2,3], Rita Maccarone[1,*] and Mauro Maccarrone [1,3,*]

[1] Department of Biotechnological and Applied Clinical Sciences, University of L'Aquila, 67100 L'Aquila, Italy
[2] Department of Medicine, Campus Bio-Medico University of Rome, 00128 Rome, Italy
[3] European Center for Brain Research (CERC)/Santa Lucia Foundation IRCCS, 00143 Rome, Italy
[*] Corresponding authors: rita.maccarone@univaq.it; mauro.maccarrone@univaq.it

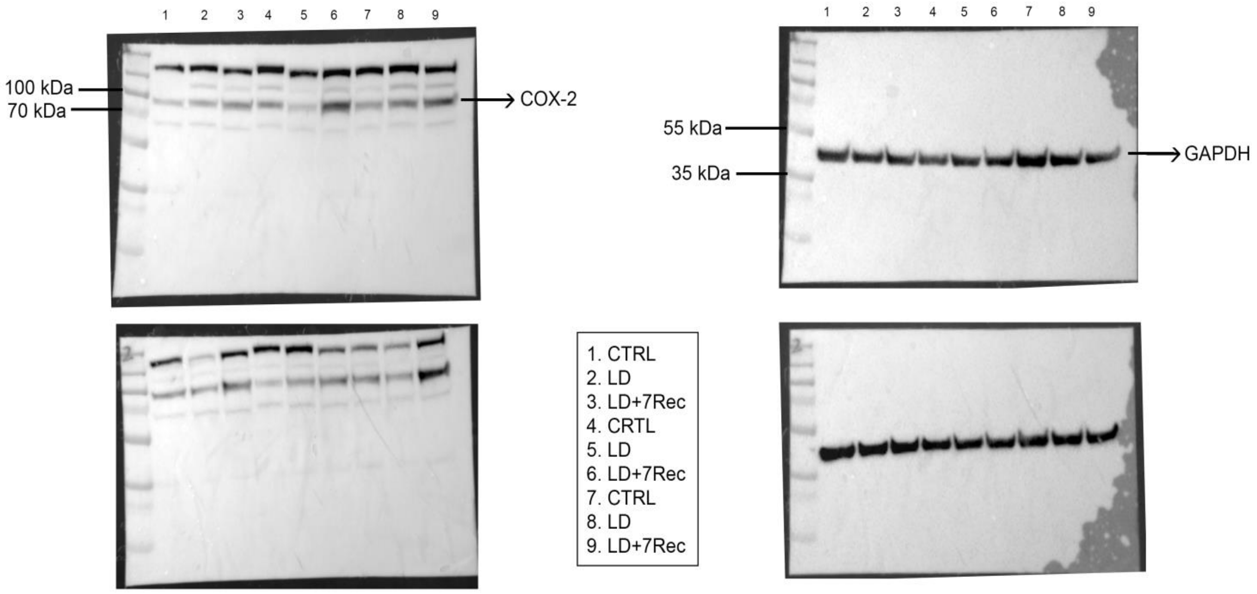

Supplementary Figure S1. Whole Western blots of COX-2 and GAPDH. Each band represents a biological replicate.

Supplementary material

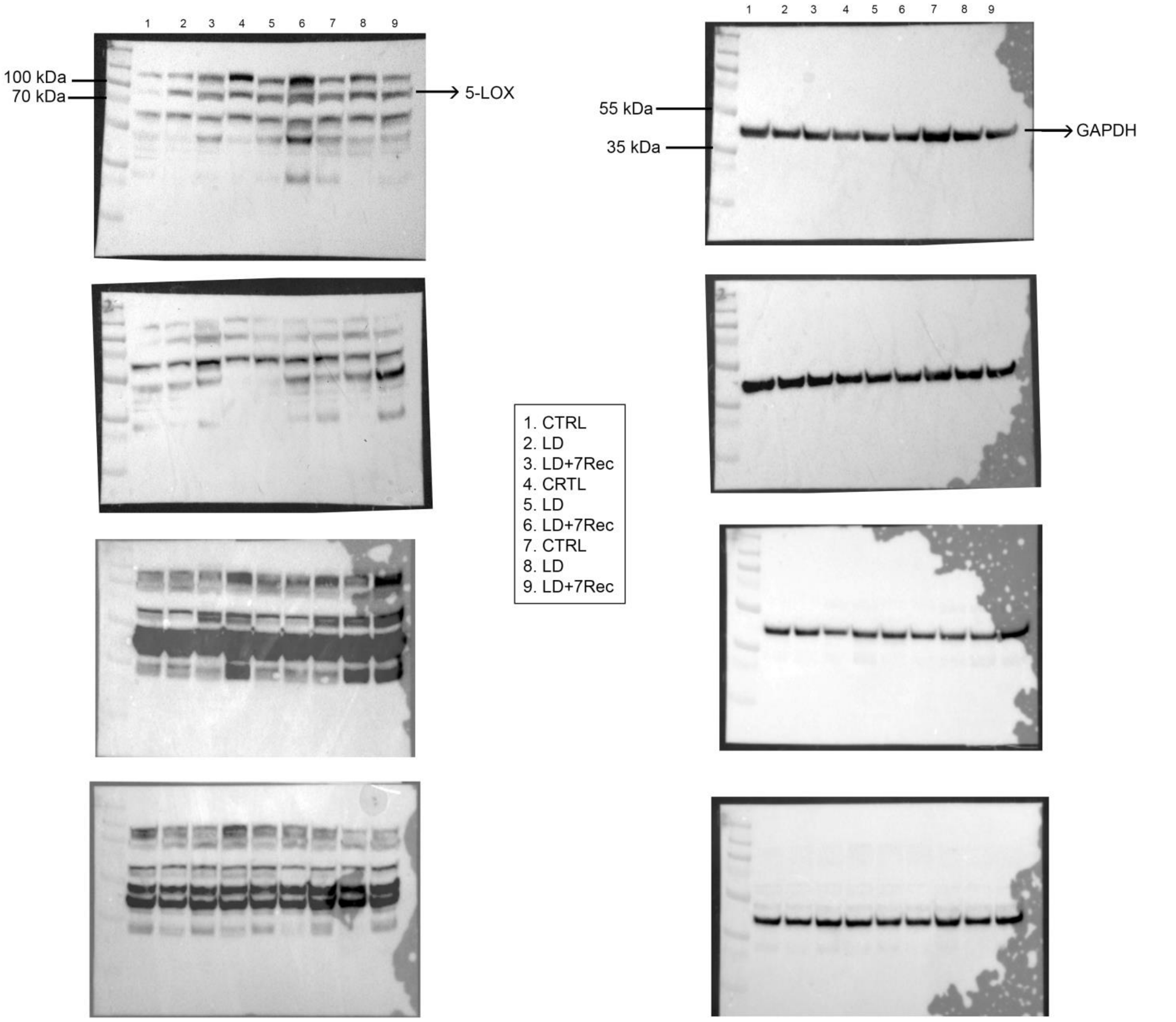

Supplementary Figure S2. Whole Western blots of 5-LOX and GAPDH. Each band represents a biological replicate.

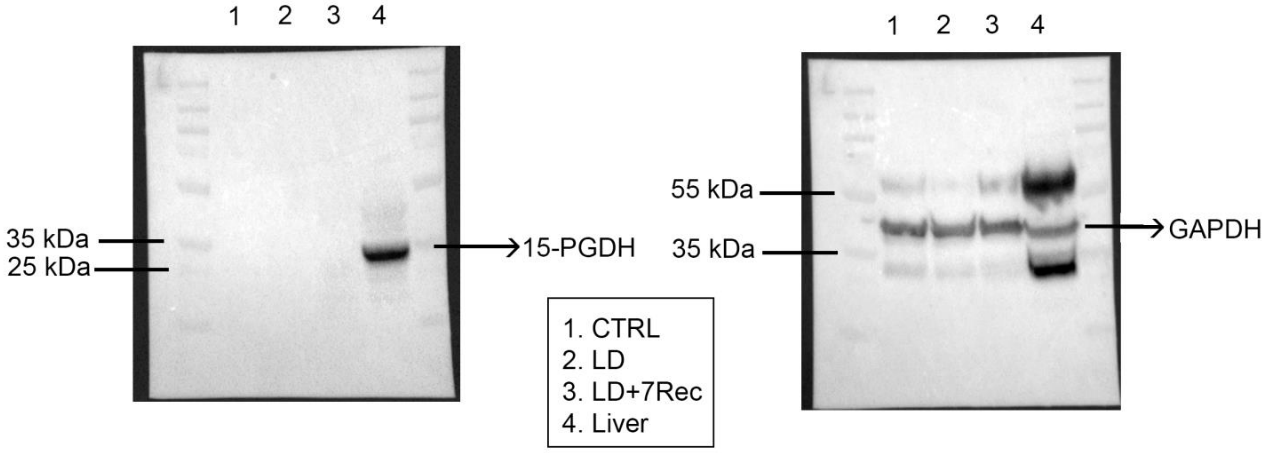

Supplementary material

Supplementary Figure S3. Whole Western blots of 15-PGDH and GAPDH.

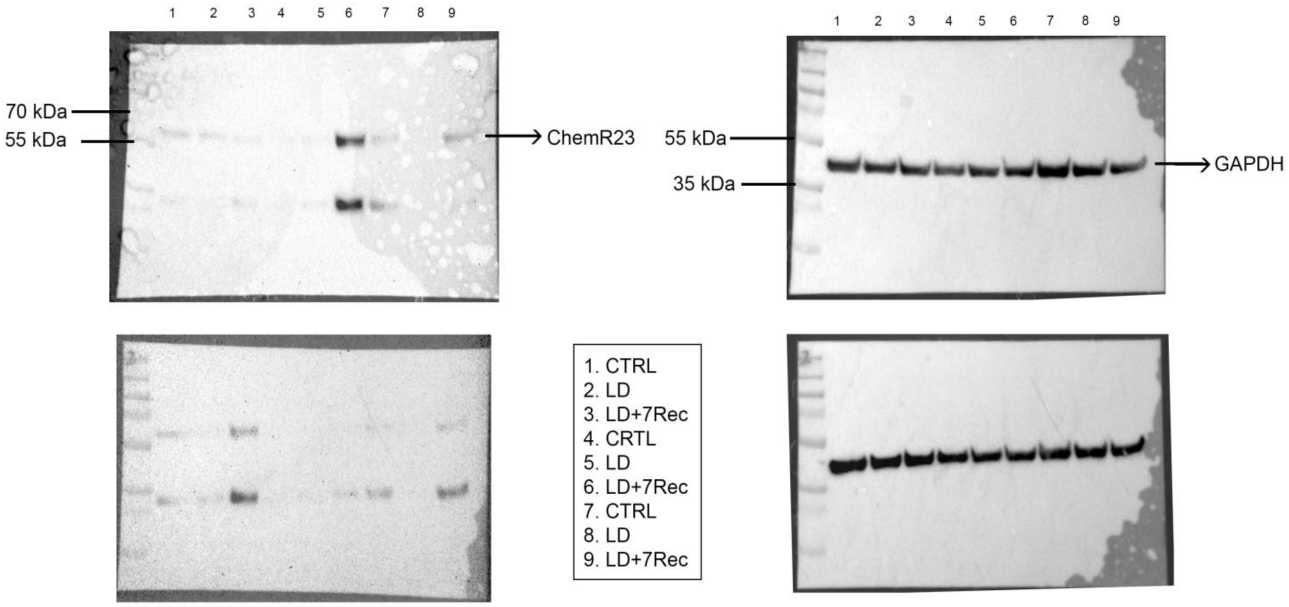

Supplementary Figure S4. Whole Western blots of ChemR23 and GAPDH. Each band represents a biological replicate.

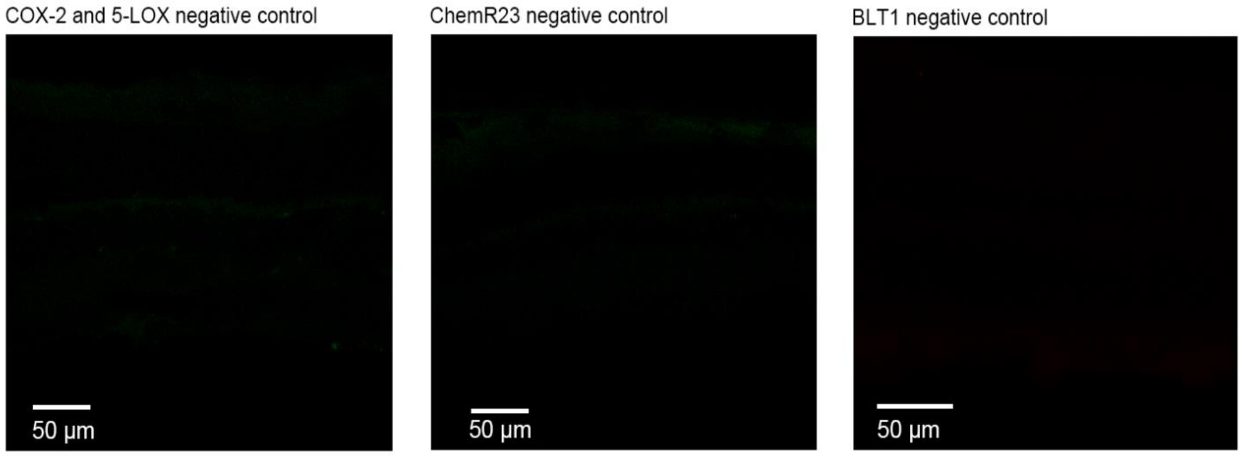

Supplementary Figure S5. Negative controls of anti-COX-2, -5-LOX, -ChemR23, and -BLT1 immunostainings. The negative control of COX-2 and 5-LOX, based on the same protocol, included the anti-mouse 488 conjugated secondary antibody. Scale bars: 50 μm.

Supplementary material

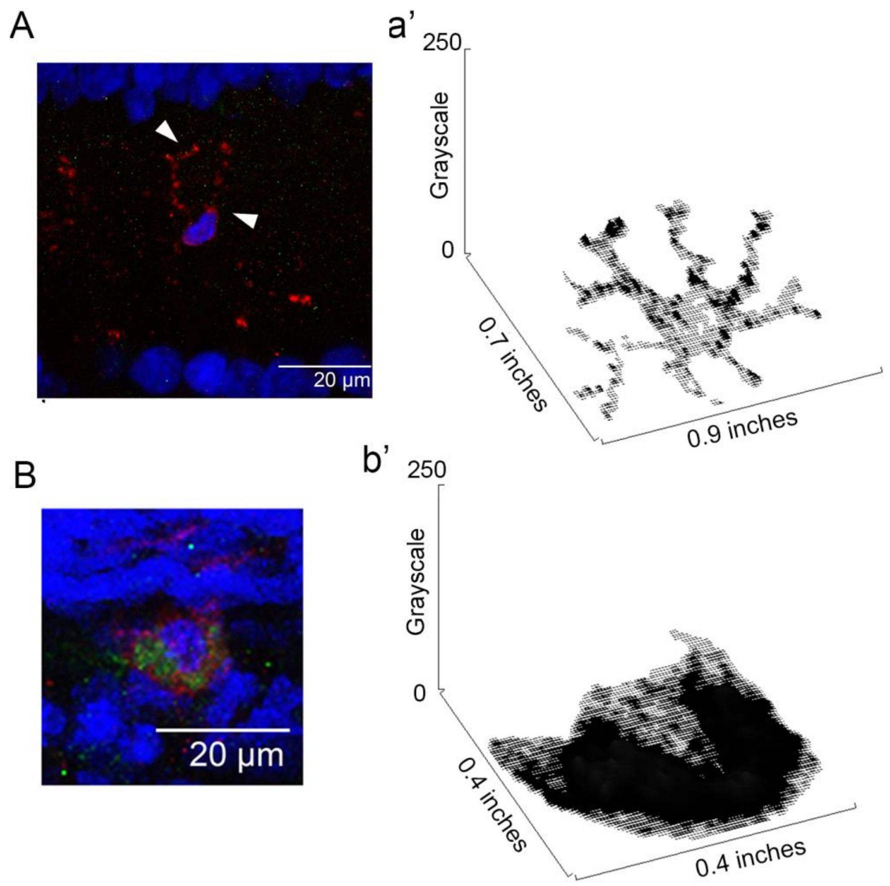

Supplementary Figure S6. Surface plot profiles of ChemR23 fluorescence in microglia. (A) Resting microglia and (a’) its surface plot; (B) activated microglia and (b’) its surface plot. ChemR23 fluorescence intensity in microglia expressed as grayscale (0-250). Green: ChemR23; Red: IBA-1; Blue: Hoechst.

Supplementary Table S1. Summary of the primary antibodies used in the study.

Antibody	Species	Company, #Catalog	Use (IF/WB)	Dilution
COX-2	Rabbit	Abcam, #ab62331	WB	1:1000
COX-2	Mouse	Santa Cruz, #sc-376861	IF	1:50
5-LOX	Mouse	Santa Cruz, #sc-136195	WB<br>IF	1:200<br>1:100
ChemR23	Mouse	Santa Cruz, #sc-398769	WB<br>IF	1:50<br>1:50
15-PGDH	Rabbit	Abcam, #ab96732	WB	1:500
GAPDH	Mouse	Invitrogen, #am4300	WB	1:3000
BLT1	Rabbit	Abcam, #188886	IF	1:100
IBA-1	Rabbit	Wako, #019-19741	IF	1:1000
